# Ecological Momentary Assessment of Illicit Drug Use Compared to Biological and Self-Reported Methods

**DOI:** 10.2196/mhealth.4470

**Published:** 2016-03-15

**Authors:** Beth S. Linas, Andrew Genz, Ryan P Westergaard, Larry W Chang, Robert C Bollinger, Carl Latkin, Gregory D Kirk

**Affiliations:** ^1^ Johns Hopkins Bloomberg School of Public Health Department of Epidemiology Baltimore, MD USA; ^2^ University of Wisconsin-Madison Departments of Medicine and Population Health Sciences Madison, WI USA; ^3^ Johns Hopkins School of Medicine and Bloomberg School of Public Health Departments of Medicine and Epidemiology Baltimore, MD USA; ^4^ Johns Hopkins School of Medicine and Bloomberg School of Public Health Departments of Medicine and International Health Baltimore, MD USA; ^5^ Johns Hopkins Bloomberg School of Public Health Department of Health, Behavior, and Society Baltimore, MD USA

**Keywords:** mHealth, ecological momentary assessment, illicit drug use, sweat patch, ACASI

## Abstract

**Background:**

The use of mHealth methods for capturing illicit drug use and associated behaviors have become more widely used in research settings, yet there is little research as to how valid these methods are compared to known measures of capturing and quantifying drug use.

**Objective:**

We examined the concordance of ecological momentary assessment (EMA) of drug use to previously validated biological and audio-computer assisted self-interview (ACASI) methods.

**Methods:**

The Exposure Assessment in Current Time (EXACT) study utilized EMA methods to assess drug use in real-time in participants’ natural environments. Utilizing mobile devices, participants self-reported each time they used heroin or cocaine over a 4-week period. Each week, PharmChek sweat patch samples were collected for measurement of heroin and cocaine and participants answered an ACASI-based questionnaire to report behaviors and drug using events during the prior week. Reports of cocaine and heroin use captured through EMA were compared to weekly biological or self-report measures through percent agreement and concordance correlation coefficients to account for repeated measures. Correlates of discordance were obtained from logistic regression models.

**Results:**

A total of 109 participants were a median of 48.5 years old, 90% African American, and 52% male. During 436 person-weeks of observation, we recorded 212 (49%) cocaine and 103 (24%) heroin sweat patches, 192 (44%) cocaine and 161 (37%) heroin ACASI surveys, and 163 (37%) cocaine and 145 (33%) heroin EMA reports. The percent agreement between EMA and sweat patch methods was 70% for cocaine use and 72% for heroin use, while the percent agreement between EMA and ACASI methods was 77% for cocaine use and 79% for heroin use. Misreporting of drug use by EMA compared to sweat patch and ACASI methods were different by illicit drug type.

**Conclusions:**

Our work demonstrates moderate to good agreement of EMA to biological and standard self-report methods in capturing illicit drug use. Limitations occur with each method and accuracy may differ by type of illicit drugs used.

## Introduction

The detection of biochemical markers of illicit drugs in biological samples of hair, urine, sweat, or blood is considered the gold standard for assessing illicit drug use and is widely used in drug treatment and employment drug testing settings [1]. The utility of these methods lies in their ability to detect metabolites of illicit drugs used within a specific window of time that varies depending on the biological specimen. Despite being the gold standard for the assessment of drug use, biological samples are often difficult to collect in the field, may be cost prohibitive, and can require greater participant engagement (eg, frequent urine screens at a treatment facility). Additionally, biologic samples typically only assess whether an individual has used drugs rather than quantifying how much and how often the drug was consumed [2,3].

In epidemiological studies, the most feasible method of assessing illicit drug use is self-report, which is recounted over extended periods of recall (eg, 6 to 12 months or longer) [4-7]. The benefit of self-report includes the ease of use, convenience, and low cost. However, whether assessed via study interviewer or audio-computer assisted self-interview (ACASI), these methods may involve recall, response, or social desirability biases [8,9]. Additionally, these methods require participants to return to the clinic or study site at regular intervals, which not only requires participants to have reliable transportation options but also disrupts their daily routines. Despite these potential issues, assessment of illicit drug use through self-report has been repeatedly shown to be a valid and reliable measure of drug use [3,10-12].

Yet, self-report and biological testing methods of capturing drug use lack the ability to assess real-time drug use, miss varying periods of intense or intermittent drug use, and cannot ascertain the proximate context of an individual’s drug using experience [13]. Ecological momentary assessment (EMA) is a mobile health (mHealth) method that is capable of collecting participant-level data in real time over notably shorter time intervals.

Mobile devices that employ mHealth strategies (eg, smartphones or other handheld devices) can utilize EMA methods for remote data collection and monitoring as well as health education and intervention [14]. EMA methods have been utilized in smoking cession studies [15-21] and among methadone-maintained outpatient drug users [22-26] but have yet to be validated as a reliable method for assessing drug use. By capturing drug-using events in real-time, outside of the study clinic and in a participant’s natural settings, a more robust, vibrant, and comprehensible understanding of drug use can be generated beyond periodic biological detection or infrequent self-reports of drug use.

Prior studies have examined concordance between the assessment of drug use by biological measures and self-report [27-29]. The aim of the current study was to investigate the concordance of assessing drug use via EMA methods compared to biological (eg, sweat patch) and ACASI methods. Additionally, we identified correlates of discordance and examined the feasibility, strengths, and weaknesses of these methods in assessing drug use among a community sample of drug users in Baltimore, Maryland.

## Methods

### EXACT Study Participants

Exposure Assessment in Current Time (EXACT) study participants were recruited from the AIDS Linked to the IntraVenous Experience (ALIVE) study, an on-going, community-recruited, observational cohort of people with a history of injecting drugs in Baltimore, Maryland [7]. The ALIVE cohort is community-based, rather than clinic-based, and thereby avoids selection bias toward people seeking or accessing health care. Details of the EXACT study have been previously described [30,31] and included 4 successive trials conducted from November 2008 through May 2013. Each trial was planned to follow 30 participants for 30 days.

Eligibility criteria for the EXACT study included current enrollment in ALIVE and the ability to understand and follow directions on a personal digital assistant (PDA) or mobile phone. Convenience sampling was utilized to identify individuals for participation in EXACT. The specific inclusion criteria regarding drug use and HIV status were varied slightly to ensure a diverse overall sample; both injection and noninjection drug users were included. Individuals were excluded if they had any medical conditions that would prevent them from operating the handheld device (eg, vision impairment) or failed to attend the screening appointment where they were trained on device use.

For Trials 1-3, participants were provided with a Palm Z22 PDA running applications developed using Satellite Forms software. All PDA programs were disabled except for study-required applications. In Trial 4, participants were provided with a Motorola Droid X2 Android mobile phone running an application developed using the electronic MObile Comprehensive Health Application platform, which was created at Johns Hopkins School of Medicine [32].

The Johns Hopkins Bloomberg School of Public Health Institutional Review Board approved the study protocol. All participants provided written informed consent. Participants were informed that involvement (or noninvolvement) in EXACT would not affect their participation in ALIVE.

### EMA Data Collection

For 30 days of observation, participants were asked to self-initiate a survey on their handheld device and self-report each time they either used heroin or cocaine (or both) in any manner (ie, smoked, snorted, or injected). For each event, participants answered questions concerning their drug use, mood, and social, physical, and activity environment, using survey instruments adapted from previous EMA studies [22-26]. To ensure responses were recorded in real-time, participants were required to indicate that drug use had occurred within 30 minutes of completing this survey. The device also delivered an end-of-day (around 9 pm) survey that asked if there was any drug use that was not reported earlier in the day. The surveys were designed to be completed in less than 3 minutes.

### Sweat Patches

PharmChek Drugs of Abuse Patches were collected weekly for the assessment of heroin or cocaine use. These patches detect traces of cocaine or heroin secreted in sweat during the period it is worn. Drugs captured via PharmChek sweat patches represent “parent” drugs (ie, same chemical compound that was taken by the drug user) and drug metabolites (ie, breakdown products of the parent drug) excreted through sweat. The patch can be worn for up to 10 days and is able to capture any drug use that occurred during the period of wear as well as 24 hours prior to patch application [33]. To ensure the patch stayed in place, an additional overlay made of the same adhesive material was worn atop the sweat patch. Once removed, patches were sent to a commercial laboratory for drug evaluation. Specimens were initially screened using an enzyme immunoassay technique with positive patches undergoing confirmation using liquid chromatography/mass spectrometry/mass spectrometry [33].

Cocaine predominates in sweat after cocaine use, however, the most common metabolite of cocaine is benzoylecgonine (BZ). A positive sweat patch result for cocaine use is confirmed by the presence of both BZ and cocaine at or above the limit of detection of 10 ng/mg. Topical analgesics, such as lidocaine or novocain, contain BZ and are used in various surgical procedures, however cocaine is structurally unique and does not resemble any of these products [33].

Opiate metabolites detectable by sweat patch include heroin, 6-monoacetylmorphine (6-MAM), codeine, and morphine. The presence of 6-MAM can only come from the use of heroin. A positive sweat patch for heroin includes the presence of the parent drug (heroin) and morphine above the limit of detection of 10 ng/ml, 6-MAM and morphine above the limit of detection of 10 ng/ml, or 6-MAM alone above the limit of detection of 10 ng/ml. The presence of morphine alone may be due to the use of other opiate-containing legal medications (eg, oxycodone, hydrocodone) or the consumption of certain foods (eg, poppy seeds). Therefore, the presence of morphine alone does not indicate a positive sweat patch for heroin [33].

### Self-Report by ACASI

Participant baseline characteristics were obtained from ACASI completed at enrollment into EXACT and/or from the prior ALIVE study visit. Additionally, at the conclusion of each study week, participants returned to the study site to answer an ACASI that included questions concerning activities, behavior, and drug use frequency during the prior week. In addition to sociodemographic variables (eg, age, sex, race, education, marital status, employment, income, homelessness, and health insurance status), baseline data collection included self-reported alcohol, tobacco, and illicit drug use; an index of drug abuse (Drug Abuse Screening Test (DAST)) [34]; and depressive symptoms (Center for Epidemiologic Studies-Depression Scale (CES-D)) in the prior 6 months [35]. Clinical characteristics (eg, HIV/antiretroviral therapy status, CD4 T-cell count, HIV RNA levels, and hepatitis B and C status) were obtained from the existing ALIVE database.

### Data Analysis

Data from all 4 trials were aggregated and analyzed together. To ensure accurate comparisons between each method of capturing drug use, all analyses were assessed by week (this was necessary as the ACASI and sweat patch data were only collected weekly). The day during which the sweat patch was placed on the participant’s arm and when the handheld device was provided represented the start of the study week 1. Seven days later, when the ACASI was completed, marked the end of the week. At this time, the sweat patch was removed and replaced with a new patch. This process was repeated for all 4 weeks of the study. Drug use reported by ACASI and sweat patch indicated use or no use within the prior week.

Real-time heroin or cocaine use, reported via the EMA entries or the end-of-day survey were summed by day and week for each participant. For analysis, an individual was considered to have used drugs if at least 1 report of drug use (eg, heroin or cocaine use by any manner) was reported in real-time within a given study week. Heroin-only and cocaine-only reports incorporated any reports of heroin or cocaine use (including those jointly used with another drug).

Because the sweat patch was able to capture drug use that included the 24 hours prior to adhesion, the EMA week was offset by 1 day to ensure concurrent periods of time were evaluated when comparing the methods. There was no adjustment for time for comparisons between EMA- and ACASI-assessed drug use.

To examine the concordance of drug use reported by EMA to sweat patch or to ACASI methods, percent agreement and concordance correlation coefficients were calculated. The concordance correlation coefficient is a measure of the level of agreement that takes into account the agreement occurring by chance for repeated measures (much like the kappa statistic is for categorical variables measured at 1 point) [36]. Using the same scale for kappa values for comparing categorical variables, concordance correlation coefficients of less than 0.2 are considered poor; 0.21-0.40 fair; 0.41-0.60 moderate; 0.61-0.80 good; and 0.81-1.00 very good [37].

If the number of EMA events in any week was greater than the number of ACASI or sweat patch responses it was considered EMA overreporting, while EMA underreporting was determined if the number of EMA reports were fewer than those reported by sweat patch or ACASI. To determine correlates of discordance between methods of assessing drug use, logistic regression models with generalized estimating equations (GEE) were examined. GEE methods adjusted for the correlation of repeated measures within each subject ID over the 4 weeks of follow-up. Variables selected for the final multivariable models were chosen through step-wise logistic regression with inclusion of significant variables (*P*<.1) from the univariate analyses. The different technologies used (eg, PDA vs mobile phone) were not accounted for analytically because the mobile platforms were very similar and we had no a priori reason to suspect that mobile platform type would in any way influence measurement. All analyses were performed using Stata release 12 (StataCorp LP, College Station, Texas).

## Results

Among 109 EXACT participants contributing 436 weeks of observation (Table 1), the median age was 48.5 years (interquartile range (IQR) 43-53 years), 98 (90%) were African American, 58 (52%) were male, and 64 (59%) were HIV infected. In the 6 months prior to baseline assessment, 23% of participants reported recent methadone treatment and 83% reported smoking cigarettes.

**Table 1:** Baseline characteristics of EXACT participants by trial^a^.

Type of Variable	Characteristics	All Trials (N=109)
		n (%)
Sociodemographic	Median age, y (IQR)	48.5 (43.3-52.9)
	African American	98 (90)
	Male	58 (52)
	Completed high school	44 (41)
	Never married	66 (61)
	Annual income < $5000	83 (78)
	Medical insurance	93 (85)
	Homeless	9(8)
		
Substance Use	Cigarettes	91 (83)
	Alcohol	71 (65)
	Marijuana	27 (25)
	Cocaine, any route	50 (46)
	Heroin, any route	49 (46)
	Speedball^b^	25 (24)
	Drug Abuse Screening Test, DAST>16	6 (18)
		
Clinical	Have primary care doctor	97 (89)
	Emergency room visit	28 (26)
	Depressive symptoms, CES-D>23	26 (23)
	Methadone treatment	26 (23)
	Hepatitis C virus seropositive	94 (86)
	HIV positive^c^	64 (59)
	Median CD4 (IQR)^c^	360.5 (239-529)
	HIV viral load > 500 copies/mL^c^	35 (55)
	Any retroviral therapy	42(65)

^a^All baseline characteristics represent behavior within the 6 months prior to the start of EXACT.

^b^A speedball is defined as the simultaneous injection of a mixture of cocaine and heroin.

^c^HIV-positive status was an inclusion criterion for Trials 3 and 4; CD4 and viral load were tested on HIV-positive participants only.

### Comparison of Methods to Capture Illicit Drug Use

Out of a possible 436 weeks of follow-up (109 individuals followed for 4 weeks), 12 weeks did not have evaluable EMA assessments of drug use, resulting in 424 weeks (97%) of observable data. Over 436 weeks, 396 (91%) sweat patches were returned and 410 (94%) ACASI surveys were completed. Missing data was the result of 22 individuals who were unable to return 29 (7%) sweat patches that were damaged or removed prematurely and 12 individuals failing to complete 14 (3%) ACASI surveys. Reports of drug use by EMA represent any report in a week and not the number of individuals or the total amount of uses in a week.

**Figure 1:**
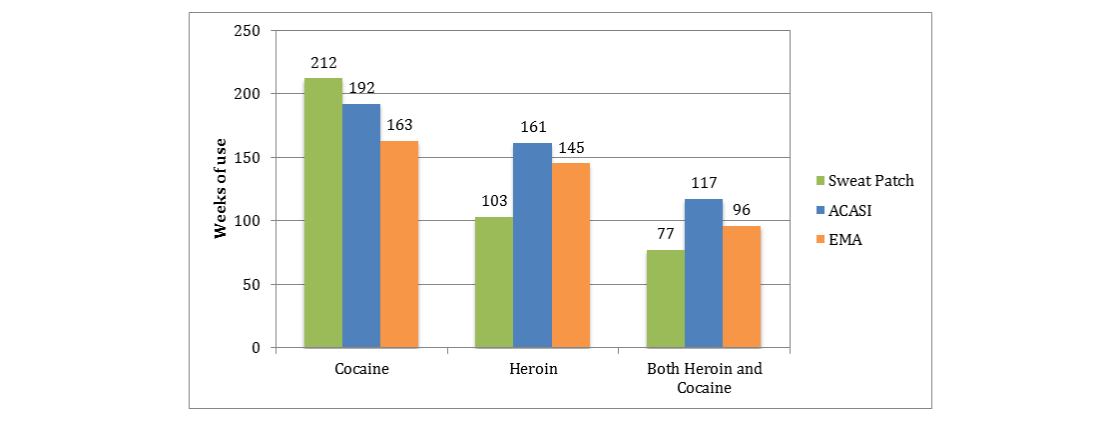
Reported drug use as assessed by sweat patch (green bars), EMA (orange bars) or ACASI (blue bars) methods.

Total weeks of drug use obtained from sweat patch, ACASI, and EMA methods are described in Figure 1. Over 436 weeks of study follow-up, 212 (49%, green bars) sweat patches, 192 (44%, blue bars) ACASI surveys, and 163 (37%, orange bars) weeks of EMA reports were positive for any cocaine use. For heroin use, 103 (24%) sweat patches, 161 (37%) ACASI surveys, and 145 (33%) weeks of EMA reports were captured over follow-up. Seventy-seven (18%) sweat patches, 117 (27%) ACASI surveys, and 96 (22%) weeks of EMA reports captured both cocaine and heroin use. The proportion of sweat patches with heroin and cocaine detected remained stable by study week.

**Figure 2:**
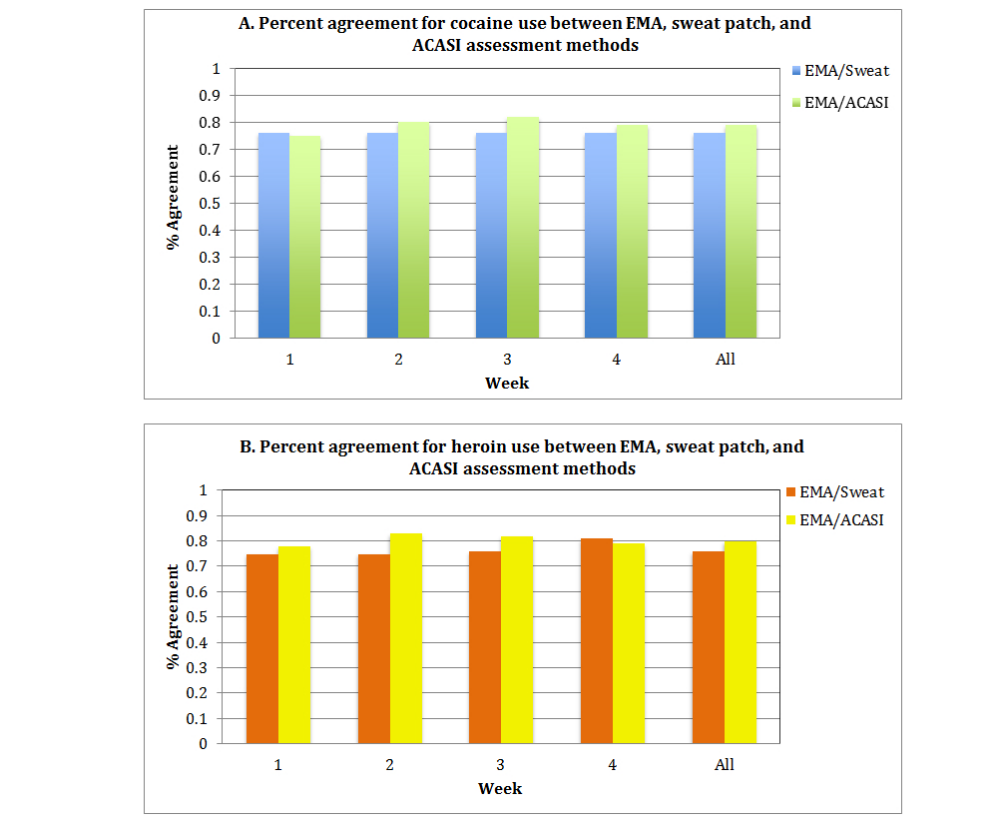
Percent agreement by drug type and week comparing EMA, sweat patch, and ACASI methods. Panel A: Percent agreement between EMA/Sweat Patch methods (blue bars) and EMA/ACASI methods (green bars) by week for cocaine use. Panel B: Percent agreement between EMA/Sweat Patch methods (orange bars) and EMA/ACASI methods (yellow bars) by week for heroin use.

For cocaine use, the overall percent agreement between EMA and sweat patch methods was 70% (Figure 2a, blue bars) (Table 2) and for EMA and ACASI methods was 77% (Figure 2a, green bars). For heroin use, the percent agreement between EMA and sweat patch methods (Figure 2b, orange bars) was 72% and for EMA and ACASI methods (Figure 2b, yellow bars) was 79%. With heroin or cocaine use, the percent agreement was slightly higher between the EMA and ACASI methods compared to EMA and sweat patch assessments.

Percent agreement does not take into consideration the agreement between 2 methods solely due to chance. The concordance correlation coefficient is a measure of inter-rater reliability that takes into account the agreement occurring by chance for repeated measures. The concordance correlation coefficients were slightly lower for comparisons of drug use between EMA and sweat patch methods than observed for EMA and ACASI methods. The concordance correlation coefficients for the comparison of EMA and sweat patch methods were in the moderate agreement range for both cocaine 0.51 (0.48-0.59) and heroin 0.48 (0.40-0.55) use. The agreement in reports between EMA and ACASI methods for cocaine use was 0.59 (0.53-0.65) and for heroin use was 0.61 (0.55-0.67), with the former representing moderate agreement and the latter representing good agreement. The agreements in reports between ACASI and sweat patch methods were not as consistent between heroin and cocaine use. The concordance correlation coefficient for the comparison of ACASI and sweat patch methods for cocaine use was 0.72 (0.67-0.77), representing good agreement, while the concordance correlation coefficient for heroin use was 0.58 (0.51-0.64), representing moderate agreement.

Misreporting of responses by EMA relative to sweat patch and ACASI methods were assessed for cocaine and heroin separately (Table 2). Relative to sweat patch results, underreporting of drug use by EMA methods was more likely for heroin than cocaine use (19% vs 9%), but overreporting by EMA methods was greater for cocaine than heroin use (21% vs 8%). Misreporting was identified less commonly between EMA and ACASI methods. Compared to ACASI reports, underreporting by EMA was infrequent and similar for both cocaine and heroin use (8% vs 9%). Overreporting by EMA relative to ACASI was slightly greater for cocaine than heroin use (15% vs 13%).

**Table 2:** Percent agreement and over- and underreporting of EMA responses compared to sweat patch and ACASI responses by drug use type^a^.

		Cocaine	Heroin
		n (%)	n (%)
Sweat Patch	Reported yes on EMA/sweat patch negative (overreport)	30 (9%)	70 (19%)
	EMA & sweat patch concordant	298 (70%)	307 (72%)
	Reported no on EMA/sweat patch positive (underreport)	79 (21%)	28 (8%)
			
ACASI	Reported yes on EMA/ACASI negative (overreport)	29 (8%)	33 (9%)
	EMA & ACASI concordant	327 (77%)	335 (79%)
	Reported no on EMA/ACASI positive (underreport)	58 (15%)	49 (13%)

^a^Sweat patches and ACASI captured both cocaine and heroin use (ie, there is double counting); therefore, numbers do not add up to 100%.

### Correlates of EMA Misreporting

We sought to identify sociodemographic, behavioral or clinical factors associated with over- or underreporting of drug use by EMA. Relative to sweat patch results, there were no significant correlates of EMA overreporting of cocaine use (Table 3). Underreports of cocaine use by EMA were almost 2-fold less likely among females (odds ratio (OR) 0.47, 95% confidence interval (CI): 0.23-0.98) and 80% less likely among individuals who reported injecting once per day or more at baseline (OR 0.21, 95% CI: 0.05-0.87). Although only marginally significant, individuals under 50 years of age were found to be less likely to underreport cocaine use as well (OR 0.51, 95% CI: 0.25-1.07).

EMA overreports of heroin use relative to sweat patches were twice as likely if heroin was used at baseline (OR 2.10, 95% CI: 1.0-4.56), but baseline heroin use was also the only factor significantly associated with EMA underreporting of heroin use (OR 5.56, 95% CI: 1.37-22.46). Female gender also achieved marginal significance in being less likely to underreport heroin use via EMA methods (OR 0.31, 95% CI: 0.08-1.10).

Compared to ACASI methods (Table 3), EMA overreports of cocaine and heroin use were twice as likely if a participant reported sharing injection needles at baseline (OR cocaine 2.79, 95% CI: 1.03-7.5; OR heroin 2.96, 95% CI: 1.17-7.51). Underreporting of cocaine use by EMA was marginally associated with being married and was 6-fold more likely if individuals reported having medical insurance at baseline (OR 6.62, 95% CI: 1.16-37.76). EMA overreports of heroin use were positively associated with baseline heroin use (OR 3.04, 95% CI: 1.33-6.95) as well as over 4-fold more likely if the participant was HIV infected (OR 4.56, 95% CI: 1.80-11.58).

**Table 3:** Correlates of misreporting cocaine and heroin use by EMA compared to sweat patch or ACASI methods^a^.

		Cocaine EMA overreport	Cocaine EMA underreport	Heroin EMA overreport	Heroin EMA underreport
		OR (95% CI)	OR (95% CI)	OR (95% CI)	OR (95% CI)
Sweat patch	Female		0.47 (0.23-0.98)^b^		0.31 (0.08-1.10)^c^
	Age≥50			0.52 (0.24-1.14)	
	Never Married		0.51 (0.25-1.07)^c^		0.68 (0.21-2.19)
	Alcohol Use		1.55 (0.72-3.36)		
	Insurance		3.15 (0.76-13.05)		
	Any heroin			2.10 (1.0-4.56)^b^	5.56 (1.37-22.46)^b^
	Any cocaine		1.55 (0.72-3.36)	1.68 (0.79-3.62)	
	Same doctor for at least 2 years			0.60 (0.30-1.23)	
	Inject≥1/day		0.21 (0.05-0.87)^b^		
					
ACASI	Age≥50			0.42 (0.17-1.07)^c^	
	Never Married		0.47 (0.21-1.02)^c^		0.53 (0.24-1.18)
	Cigarette use		2.73 (0.64-11.72)	2.88 (0.61-13.57)	2.76 (0.46-16.55)
	Alcohol use		1.73 (0.69-4.29)		
	Insurance		6.62 (1.16-37.76)^b^		
	Any heroin			0.77 (0.29-1.99)	3.04 (1.33-6.95)^b^
	Any cocaine		1.78 (0.76-4.20)	1.86 (0.77-4.51)	1.19 (0.53-2.68)
	HIV infected	0.52 (0.18-1.5)			4.56 (1.80-11.58)^b^
	Shared needles	2.79 (1.03-7.5)^b^		2.96 (1.17-7.51)^b^	
	Primary care physician	0.57 (0.15-2.14)			
	Yearly income<$5000			1.69 (0.45-6.34)	

^a^Correlates included in multivariable models had *P*<.1 in univariate analyses and represent behaviors occurring within the 6 months prior to the start of EXACT.

^b^Statistical significance, *P*<.05.

^c^Marginal statistical significance, *P*<.1.

## Discussion

This analysis demonstrated moderate to good concordance and inter-rater reliability of reported drug use by EMA when compared to either biological measures of sweat patches or more conventional ACASI self-report methods. However, our data raised concerns regarding the use of sweat patches as a gold standard for drug use assessment due to the notably lower prevalence of heroin use defined by biological detection compared to the prevalence of heroin use we determined in this study based on self-reported methods and to the expected prevalence based on prior data in our ALIVE cohort [7,38]. Even relative to imperfect gold standards, we provide evidence that researchers should be confident that EMA methods can accurately capture and characterize illicit drug use comparable to currently used methods. Given the relative benefits of daily real-time assessments of drug use in terms of reductions in recall bias, social desirability bias (reductions in participant need to please the interviewer), participant burden, and follow-up time, EMA methods for assessing drug use may have broad applications in settings ranging from epidemiological studies to behavioral interventions.

### EMA Compared to Sweat Patch Assessment

Biological samples serve as the gold standard for assessing drug use because they are able to capture the biochemical components of drug use as the body excretes them. Sweat patches are often used to detect longer-term drug use and can continuously capture drug metabolites in sweat until the patch is removed. It is designed to be flexible, waterproof, and safe from environmental contaminates [39]. However, once the patch comes off the skin, it cannot be put back on to resume drug use capture. Current applications of sweat patch testing include use in drug treatment for monitoring drug relapse and for determining the effectiveness of medical and psychological therapy [40,41].

Our results suggest moderate concordance between EMA and sweat patch methods for assessing drug use. Prior studies have shown substantial discordance between self-report of drug use and biochemical tests results across out-of-treatment populations [42]. A 10-week outpatient clinical trial in which participants wore sweat patches and provided urine samples and self-reports of cocaine use thrice weekly demonstrated the concurrent validity of urine and sweat patches to be reasonable (correlation: 0.76, *P*<.001), but the correlation between self-report and the patches was lower (correlation: 0.40, *P*<.05) [43]. A separate outpatient study examining the utility of sweat testing for monitoring drug use also found the level of agreement between positive sweat test results and positive urine results to be 33% for heroin and 92% for cocaine [40].

The results of our sweat patch analyses demonstrated a notably greater number of cocaine-positive sweat patches compared to heroin-positive sweat patches. This finding was unexpected as our prior analyses with this EXACT population demonstrated heroin to be the predominant drug of use over 30 days of follow-up [30]. Additionally, recent estimates indicate that Baltimore suffers from a far greater public health burden of heroin abuse compared to cocaine abuse [44] and this is mirrored in the participants of the ALIVE study [7,38]. Upon consultation with PharmChek*,* manufacturers of the sweat patches, it was suggested this difference may have been the result of our heroin-using participants using such small amounts of heroin that, even after a week of wearing the patch, they did not secrete enough heroin metabolites to be detected at the limit of detection of 10 ng/ml (Matthew Hartley, personal communication).

Although concerns have been raised that sweat patches may serve as a deterrent to drug use [33], there was no incentive in this study to modify behavior and our self-reported drug use data did indicate greater heroin use. To explain our findings, there would have to be a differential effect resulting in heroin misreporting relative to cocaine in response to sweat patch placement, which seems implausible. Yet, despite these problems of relatively lower heroin detection via sweat patches, the inter-rater reliability of EMA methods compared to sweat patch analysis remained moderate for both heroin and cocaine use as evidenced by concordance correlation coefficients.

### EMA Compared to ACASI Assessment

ACASI methods have now become the standard approach for collecting sensitive data in epidemiologic research studies. The use of ACASI has resulted in greater disclosure of sensitive behaviors such as drug and sexual risky behaviors in some population [45-47], thereby reducing social desirability bias and improving accuracy of self-report.

Although the best time interval for assessing drug use exposure remains unknown, several studies have found that reporting sensitive sexual behaviors can be accurately recalled for intervals of 1-3 months [48,49]. Longer time frames may be more representative of a person’s behavior patterns, but can be more difficult to recall. It is likely that participants asked to recall behaviors over longer time periods may rely on a strategy such as “guestimation” of the average number of days per week they have been with a specific partner or used drugs [50]. Despite the potential problems with accuracy of information collected over longer periods of time, ACASI assessments are rarely done in shorter intervals due to practical issues.

In this analysis, the inter-rater reliability and concordance of EMA methods compared to ACASI methods for assessing drug use appear stronger for heroin use (concordance correlation coefficient for heroin use had good agreement, 0.61 (0.53-0.69)) than for cocaine use. Both methods involve self-report in settings with increased privacy over traditional face-to-face interviews providing greater anonymity when disclosing sensitive information. In the current analysis, the ACASI reports captured more drug use than EMA methods. It is hard to differentiate between “fuzzy” recall that may have been reported via ACASI (leading to overreports) from participants that may have been impaired from drug use when answering the EMA survey (leading to underreports).

While we document good concordance between EMA methods with both ACASI and sweat patch approaches, this analysis neglects to consider a primary analytical strength of EMA methodology, namely the examination of real-time drug use. EMA methods allow for the examination of the variation and amount of drug use by day. ACASI methods are unable to examine daily drug-using patterns due to feasibility issues (eg, study visits, etc). Despite these differences in methodologies, our EMA results remained reliable when compared to ACASI-based methods and could prove extremely useful in understanding drug use among chronic drug users.

### Misreporting by EMA

Relative to sweat patch reports, there were no demographic or behavioral correlates of overreporting of cocaine use by EMA whereas having a stable partner, male gender, and daily injection at baseline were associated with underreporting of cocaine use by EMA. Baseline heroin use was the only significant correlate of misreporting of heroin by EMA relative to the sweat patch. In total, these data suggest that more regular heroin users were more likely to both overreport and underreport heroin, raising concerns regarding misclassification due to the limitations of sweat patch detection of heroin as highlighted above. In-depth or timeline follow-back interviews could be used to better understand discordant events, as we do not know if the dynamics of overreporting and underreporting are the same.

Relative to ACASI, sharing injection needles was positively associated with EMA overreporting of both cocaine and heroin use, while underreports of cocaine use was positively associated with having medical insurance. In contrast, EMA underreporting of heroin use were positively associated with baseline heroin use and HIV status. Stable factors, such has having insurance, may lead to underreporting of cocaine use as a result of a social desirability bias. The associations of misreporting with sharing needles, baseline heroin use, and HIV status most likely reflects the recruitment criteria of EXACT, which included a large proportion of HIV-infected individuals with more recent and intense drug use.

### Conclusions

Substance abuse is commonly associated with a chaotic or disordered life, mental illness, financial and legal difficulties, and inadequate housing or transportation [51-53]. As members of the ALIVE cohort, all EXACT participants had a history of abusing illicit drugs and most were HIV infected. Long-term chronic drug abuse has physical ramifications but also cognitive effects. Working memory deficits are also prominent among HIV-infected individuals. During periods of intoxication, heroin users suffer a slow drop-off in attention and often fall asleep [54]. Baseline cocaine and heroin use heavily impacted EMA misreporting of heroin use but not cocaine use. It is possible that inattention or sleep may have contributed to heroin users (more so than cocaine users) having difficulties in recalling drug use on a weekly basis (both as overreports and underreports) as was required of drug use assessed by ACASI. With respect to EMA underreporting, we found no evidence of exhaustion from using the handheld devices in reporting drug use across study week or by drug type. Notwithstanding these differences in reporting by drug type, EMA methods captured much of the drug use reported by ACASI methods.

There are several strengths of EMA methods that make them desirable to capture illicit drug use among community-dwelling populations. Because they are assessed in essentially real-time, EMA does not require individuals to recall or remember behaviors for prolonged periods. Social desirability bias is a reporting bias that arises when individuals underreport specific behaviors or actions because they believe these actions are sensitive and not socially acceptable to report [55]. ACASI methods have been shown to decrease social desirability bias by allowing greater respondent privacy since questions are administered audibly and in text on a computer screen in a private room without the direct participation of a study interviewer [46]. However, EMA methods may allow for even greater respondent privacy since participants are able to answer questions in their natural environment, which allows participants to calmly respond to questions where they feel most comfortable, away from a study site. ACASI interviews and sweat patches require participants to visit study sites at regular intervals.

EMA methods can provide more intensive follow-up opportunities compared to sweat patch or ACASI assessment. Daily outcome assessments over extended periods of time are feasible when using EMA methods because participants can carry the devices 24 hours a day, 7 days a week. Prior studies have involved participants using the devices for up to 6 months [24]. Real-time data capture allows for the daily context and “situatedness” of drug use to be assessed, including information on the number of days used, frequency and amount used in a day, as well as participant behaviors that occur due to specific cultural, organizational, and structural environments [13]. Historically, ACASI has resulted in greater disclosure of sensitive information [45-47] and previous EMA analyses by ourselves and others have demonstrated that EMA is capable of capturing this type of information as well [23,25,26,30,56].

Assessing drug use in epidemiologic studies ideally involves an approach that is unobtrusive, does not rely on recall, has limited requirements for participant participation, and is accessible and affordable. mHealth may provide an excellent solution for assessing drug use in the field. The level of concordance between EMA and traditional biological and self-report methods suggests that utilizing EMA mHealth strategies are feasible for assessing drug use among community dwelling, nontreatment-seeking drug users. Future studies integrating EMA methods with the use of sensors for assessing drug use will likely provide both the biological and environmental cues of illicit drug use as well as provide a more complete picture of drug-using behaviors.

## Abbreviations

ACASI: audio-computer assisted self-interview
ALIVE: AIDS linked to the intravenous experience
BZ: benzoylecgonine
CES-D: Center for Epidemiologic Studies-Depression Scale
CI: confidence interval
DAST: drug abuse screening test
EMA: ecological momentary assessment
EXACT: Exposure Assessment in Current Time
GEE: generalized estimating equations
IQR: interquartile range
mHealth: mobile health
OR: odds ratio
PDA: personal digital assistant
6-MAM: 6-monoacetylmorphine
